# Room Temperature Synthesis of a Novel Quinolinoxazine, Polymerization and Flammability Studies

**DOI:** 10.3390/polym17182546

**Published:** 2025-09-20

**Authors:** Maria Laura Salum, Daniela Iguchi, Carlos Rodriguez Arza, Nora Pellegri, Hatsuo Ishida, Pablo Froimowicz

**Affiliations:** 1 Department of Macromolecular Science and Engineering, Case Western Reserve University, Cleveland, OH 44106, USA; mls323@case.edu (M.L.S.); caroarza@gmail.com (C.R.A.); 2 CONICET, Instituto de Física Rosario (IFIR), Universidad Nacional de Rosario, 27 de Febrero 210 Bis, 2000 Rosario, Argentina; iguchi@ifir-conicet.gov.ar (D.I.); pellegri@ifir-conicet.gov.ar (N.P.)

**Keywords:** quinolinoxazine, benzoxazine, room temperature synthesis

## Abstract

A novel quinoline-containing benzoxazine resin, **8HQ-fa**, has been successfully synthesized at room temperature using sustainable raw materials, such as 8-hydroxyquinoline and furfurylamine as the phenol and amine source, respectively. The chemical structure of the hereinafter referred to as quinolinoxazine is fully characterized by Fourier transform infrared spectroscopy (FT-IR), ^1^H and ^13^C nuclear magnetic resonance spectroscopy (NMR), as well as by 2D ^1^H–^1^H nuclear Overhauser effect spectroscopy (NOESY) and ^1^H–^13^C heteronuclear multiple quantum correlation (HMQC) NMR. Thermal properties and polymerization behavior of the monomer are studied by differential scanning calorimetry (DSC) and thermogravimetric analysis (TGA). The resulting polymer is also characterized in terms of its thermal and fire-related properties by DSC, TGA, and microscale combustion calorimetry (MCC). The resulting thermoset, **poly(8HQ-fa)**, presents good thermal stability as evidenced by its *T*_g_ (201 °C), *T*_d5_ and *T*_d10_ (307 and 351 °C, respectively), and char yield (42%), and low flammability as determined by the LOI, heat release capacity, and total heat released values (34.3, 143 J/gK, and 10.8 kJ/g, respectively), making it a self-extinguishing thermoset. The combination of properties and advantages in the synthesis of **8HQ-fa**, accompanied by a low polymerization temperature, suggests its great potential in the field of high-performance polymers.

## 1. Introduction

From its inception, the field of polybenzoxazines has been recognized by academia and industry not only as an attractive alternative to other traditional thermosets but also as an interesting new class of material. The diversity of desirable properties exhibited by polybenzoxazines makes it easy to understand how a polymer with such a short history has already gained its place in the industrial market, including very rare commercialization of the new resin. Polybenzoxazine is produced by polymerizing 1,3-benzoxazine resins [1]. These monomeric benzoxazines offer an extraordinary flexibility in their molecular design, thus being able to tailor desired properties into the final polymer materials [2]. For instance, the most basic benzoxazine, which is synthesized from phenol, formaldehyde, and aniline (known as PH-a), has eleven positions where potential substituents may be introduced. Moreover, both moieties, phenol and aniline, are frequently replaced by other compounds or generic families of compounds providing phenol-like –OH groups, such as cardanol [3,4], eugenol [3,5], coumarin [6], vanillin [7,8], guaiacol [9,10], resorcinol [11,12], diphenolic acid [13,14], and phloretic acid [15,16], amine groups such as methylamine [17,18], hexylamine [19], stearylamine [20,21], furfurylamine [6,20], as well as polymeric amines like jeffamines [22,23] and chitosan [24,25]. These efforts have recently been reviewed [26,27]. However, despite having this possibility for diversifying the chemistry and properties, the field of polybenzoxazines faces similar challenges as many other polymers regarding accessibility and availability of novel raw materials, costs, and even philosophical bent targeting, for example, green and/or sustainable approaches. In this regard, much effort has been invested in generating bio-based macromolecular materials, although the use of industrial waste as raw materials seems not to have been exploited as much yet. An inspiring example of the latter has been reported by Pyun and co-workers [28], where elemental sulfur has been cleverly utilized as feedstock to generate high-end co-polymeric materials. Based on the approach followed, which briefly consisted of using elemental sulfur as the major co-monomer in reacting systems with vinyl monomers and crosslinkers, the methodology is now known as inverse vulcanization and is currently successfully exploited in different fields.

Similarly to the case of elemental sulfur in the petroleum industry, coal tar is a byproduct in the production of coke [29] and coal [30]. Coal tars are, in general, complex mixtures of different polycyclic aromatic compounds, such as quinolines [30]. Coal tar is, in fact, one of the main sources for commercially obtaining quinoline, which is the starting material for synthesizing 8-hydroxyquinoline. Although there are other industrial methods for synthesizing 8-hydroxyquinoline, they require specific reactants and synthetic steps. Thus, revalorization of the waste generated in the production of coke and coal toward producing 8-hydroxyquinoline is a more sustainable manner. The latter statement may especially gain value if the 8-hydroxyquinoline is proven to be a useful raw material for obtaining novel material. This compound bears a phenolic –OH group with a free *ortho* position, which makes it a good starting material for the synthesis of benzoxazine-like resins. To complete the synthesis, an amine compound must also be used. With the objective of maintaining the use of sustainable raw materials, furfurylamine is proposed. Furfurylamine is produced in different ways, although it is generally from furfural [31]. Furfural, in turn, can be obtained from sugar beet [32], which would make this reactant bio-based. This same compound can also be obtained from the corncob [33], which is waste in the agricultural industry. Therefore, the production of furfurylamine, to some extent, can be considered as bio-sustainable.

Sustainable syntheses can be achieved following sustainable procedures and not necessarily relying only on the starting materials or starting materials source [34]. Thus, for example, it should be encouraged the possibility of using more benign auxiliaries in chemistry as well as conducting chemical reactions at room temperature as recommended by the less popular principles number 5 and 6 of the Twelve Principles of Green Chemistry [35]. Both strategies have been proven possible in the past [36,37], although they, in general, receive less attention than principle number 7, use of renewable feedstock.

There is a limited number of quinoline-based benzoxazines reported hitherto [19,38,39]. However, to the best of our knowledge, this is the first report combining a sustainable synthesis of a quinoline-based benzoxazine from various different perspectives and the exploration of a feasible application of its thermoset. Thus, we present in this work a sustainable room temperature synthesis of a novel benzoxazine-like monomeric resin, polymerization study, and possible application as a self-extinguishable polymer material. Last but not least, following an analogy to the known naphthozaxines [40,41], quinoline-based benzoxazines might be viewed as an entire family of compounds and therefore could be referred to as quinolinoxazines.

## 2. Materials and Methods

### 2.1. Materials

8-Hydroxyquinoline (purity > 98%) was purchased from Sigma-Aldrich (St. Louis, MO, USA) and used as received. Paraformaldehyde (≤96%) was purchased from Aldrich Chemical Co., St. Louis, MO, USA, and was used as received. 1-(2-Furyl)methylamine (referred to as furfurylamine) (98%) was used as received from TCI America (Portland, OR, USA). Acetonitrile was obtained from Fisher Scientific Company, Hampton, NH, USA, and was used as received (99%).

### 2.2. Synthesis of 3-(Furan-2-ylmethyl)-3,4-dihydro-2H-[1,3]oxazino[5,6-h]quinoline (Hereinafter Referred to as ***8HQ-fa***)

In a 50 mL round-bottomed flask, 8-hydroxyquinoline (7.4 mmol), furfurylamine (7.4 mmol), formalin (37%, *w*/*v*, 15 mmol), formic acid (0.1 mL), and acetonitrile (11 mL) were mixed and magnetically stirred at room temperature for 12 h. Ethyl acetate (50 mL) was added to the reaction mixture, and the organic phase was washed three times with H_2_O, dried over anhydrous MgSO_4_, and filtered. After removal of the solvent, a white crystalline powder was obtained, which upon recrystallization in toluene afforded white needle-like crystals. Reaction yield: 87%.

### 2.3. Characterization of ***8HQ-fa***

^1^H NMR (500 MHz, CDCl_3_): *δ* = 8.92 (dd, *J* = 4.2, 1.7 Hz, 1H), 8.10 (dd, *J* = 8.3, 1.7 Hz, 1H), 7.42 (m, 2H), 7.35 (d, *J* = 8.4 Hz, 1H), 7.14 (d, *J* = 8.4 Hz, 1H), 6.34 (dd, *J* = 3.2, 1.8 Hz, 1H), 6.27 (d, *J* = 3.1 Hz, 1H), 5.20 (s, 2H), 4.18 (s, 2H), 4.02 (s, 2H). ^13^C NMR (126 MHz, CDCl3): *δ* = 151.59, 149.70, 149.42, 142.83, 139.44, 136.12, 128.28, 126.24, 121.46, 119.42, 117.61, 110.35, 109.31, 82.92, 49.65, 48.66. FT-IR (KBr): *ν* (cm^−1^) = 1246, 1180, 1126, 1080, 1028, 916, 853.

### 2.4. Preparation of Polybenzoxazine

The quinolinoxazine resin, **8HQ-fa**, was polymerized following a three-step polymerization process, consisting of isothermal heating steps at 207, 223, 239 °C for 3 h each. The polymerization process was based on the differential scanning calorimetry (DSC) results.

### 2.5. Instrumentation

Fourier transform infrared (FTIR) spectra were recorded using a Bruker INVENIO-X Fourier (Bruker, Billerica, MA, USA) transform infrared (FT-IR) spectrometer. Absorption spectra were recorded employing KBr pellets, co-addition of 16 scans at a resolution of 4 cm^−1^. ^1^H and ^13^C NMR spectra were recorded at a proton frequency of 500 MHz and the corresponding carbon frequency of 125.721 MHz, respectively, using deuterated chloroform-*d* (CDCl_3_) as solvent, in a Bruker Avance spectrometer (Bruker, Billerica, MA, USA). Two-dimensional NMR spectra, ^1^H–^13^C heteronuclear multiple quantum correlation (HMQC), and two-dimensional ^1^H–^1^H nuclear Overhauser effect spectroscopy (NOESY), were recorded in the same instrument. Differential scanning calorimetry (DSC) measurements were carried out using a TA Instruments Discovery DSC 250 (TA Instruments, New Castle, DE, USA) under a N_2_ atmosphere at a flow rate of 50 mL/min. Typically, nonisothermal measurements at a heating rate of 10 °C/min from 30 to 300 °C were applied. All samples were prepared in sealed aluminum pans. Measurements carried out for the activation energy determination used heating ramp rates of 5, 10, 15, 20, and 25 °C/min. Both the Kissinger and modified Ozawa equations were used to calculate the activation energies [42,43,44]. Thermogravimetric analysis (TGA) measurements were carried out using a TA Instruments TGA500 (TA Instruments, New Castle, DE, USA), under a N_2_ atmosphere at a flow rate of 60 mL/min. A heating ramp rate of 10 °C/min from 30 to 800 °C was applied. The char yield was defined as the residual weight, in percentage, at 800 °C under inert atmosphere. Platinum pan was used in all cases. Heat release capacity (HRC), total heat release (THR), and fire growth capacity (FGC) were measured using a microscale combustion calorimetry (MCC), Deatak model MCC-4 (Deatak, McHenry, IL, USA). Typically, measurements were carried out from 75 to 750 °C at 1 °C/s heating rate under a stream of 80/20 volume ratio of N_2_:O_2_ atmosphere.

## 3. Results and Discussion

**Scheme 1:**
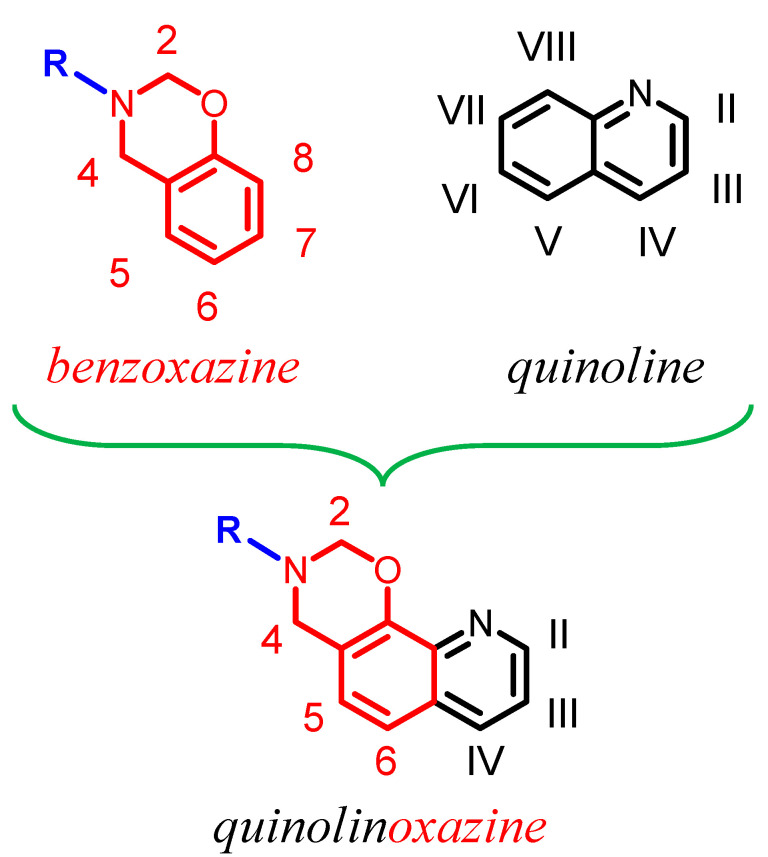
Typical accepted numbering in benzoxazine (red) and quinoline (black) nuclei identifying each position. In the chemical structure of the so-called “*quinolinoxazine*” (bottom), Arabic numbers indicate positions following the benzoxazine nomenclature, while Roman numbers denote positions in the quinoline portion not overlapped with the benzoxazine structure. The remaining amine portion (in blue) of the molecule is omitted at this time to gain simplicity.

The synthesis of monomeric benzoxazine resins can generally be carried out by following several different experimental procedures modifying the typical Mannich reaction [6]. Nevertheless, with the right combination of phenolic –OH containing groups, amines, and formaldehyde, benzoxazines with specific chemical structures can be synthesized with customized properties, regardless of the experimental procedures chosen. As stated in the Section 1, the primary interest in our molecular design and experimental procedure resided in simultaneously accomplishing multiple, seemingly incompatible, objectives. First, revalorization of waste as valuable raw materials to synthesize benzoxazine monomers. In order to fulfill this, 8HQ was employed as the phenol-like compound and furfurylamine as the complementary aliphatic primary amine. Second, the reduction in the necessary energy is commonly utilized to synthesize benzoxazine monomers. With the aim of saving energy, a room-temperature strategy was chosen to be studied and compared to other more common high-temperature syntheses. Third, produce monomers that, after polymerization, will generate materials with highly desirable properties toward potential applications. In this regard, we aimed to prepare a polymer that will exhibit self-extinguishing properties. Last, and most importantly, we targeted the possibility of combining all previous individual objectives at once. As mentioned, 8HQ is one of the compounds chosen, which belongs to the larger family of quinolines. Like any other family of compounds, the quinolone family bears a common and known nucleus, with its individualized position on the chemical structure. A similar situation occurs with benzoxazines. Therefore, it is important to identify each molecular nucleus from each type of compound, to then understand not only the nomenclature used in this work but also the chemical aspects of the synthesized product, that is, the benzoxazine monomer. Scheme 1 shows the numbering identifying each individual position on each molecular nucleus, benzoxazine, quinoline, and the resulting quinoline-based benzoxazine. As can be seen, each nucleus is numbered according to its own conventional nomenclature, assigning Arabic numbers to the benzoxazine nuclei and Roman ones to the quinoline. To prevent any possible misinterpretation, the numbering system of each nucleus is respected on the resulting quinoline-based benzoxazine.

**Scheme 2:**
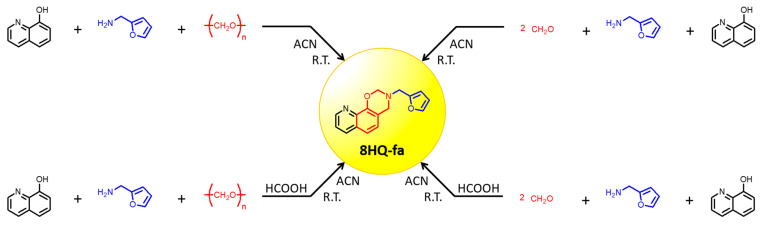
Synthesis of the benzoxazine monomer, also referred to as quinolinoxazine **8HQ-fa**.

To achieve the mentioned complex objective, the synthetic pathway presented in Scheme 2 was designed to obtain the desired quinolinoxazine. As seen in the scheme, furfurylamine was utilized as the amine, while the formaldehyde source was evaluated by using paraformaldehyde or formalin under different reaction conditions. As mentioned in the introduction, we were interested in demonstrating the feasibility of carrying out this synthesis at room temperature. Compared to commonly refluxing conditions, the reaction under room temperature conditions would be more energetically efficient and, importantly, safer since the loss of formaldehyde caused by the heating might be minimized or even eliminated. As presented in Scheme 2, four different reaction conditions were evaluated, with all of them at room temperature. The results are summarized in Table 1.

The results shown in Table 1 indicate that the best reaction condition to obtain **8HQ-fa** is that in which formalin was used as the formaldehyde source with a small addition of formic acid to the reaction medium. A reaction yield of 87% was achieved for an overnight reaction at room temperature.

### 3.1. FT-IR Characterization of ***8HQ-fa***

**Figure 1:**
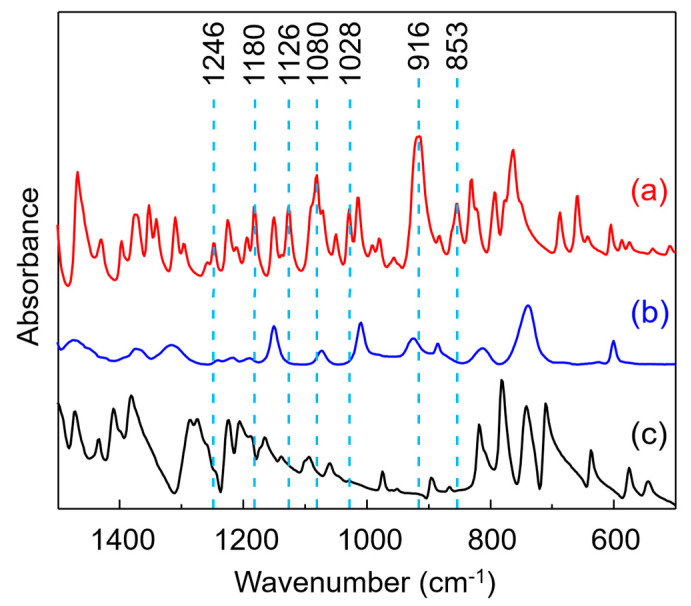
Expanded region between 1500 and 500 cm^−1^ of the FT-IR spectra of the quinolinoxazine monomer, (a) **8HQ-fa**, (b), furfurylamine, (c) and 8-hydroxyquinoline.

FT-IR spectroscopy was used to evaluate the progress of the chemical reaction to form the targeted quinolinoxazine. The expanded region between 1500 and 500 cm^−1^ of the FT-IR spectrum of **8HQ-fa** is shown in Figure 1, where the spectra of furfurylamine and 8-hydroxyquinoline are also presented for comparison.

Formation of the desired product, **8HQ-fa**, is indicated by the presence in spectrum a (Figure 1) of the bands listed and assigned in Table 2, which are not present in spectra b and c corresponding to furfurylamine and 8-hydroxyquinoline, respectively.

### 3.2. NMR Characterization of ***8HQ-fa***

**Figure 2:**
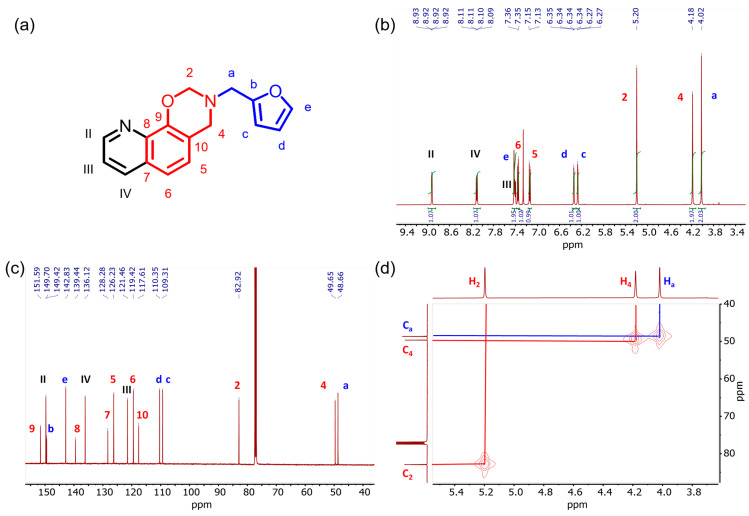
(**a**) Full numbering and lettering for identifying each position on the chemical structure of **8HQ-fa**. (**b**–**d**) ^1^H, ^13^C, and 2D ^1^H–^13^C HMQC NMR spectra of **8HQ-fa**.

NMR spectroscopy was used to gain detailed information on the chemical structure of the synthesized quinolinoxazine monomer, **8HQ-fa**. Due to the chemical structure presented by the resulting product, **8HQ-fa**, it is possible to consider it as both a quinoline-containing benzoxazine or a benzoxazine-containing quinoline. **8HQ-fa** will be considered hereinafter as a benzoxazine derivative just because of the scope of the present work. Therefore, the numbering adopted to identify each molecular position agrees with the widely established benzoxazine convention. Thus, Figure 2 shows the NMR spectral analysis, in which the numbering, including the lettering for the amine portion of the molecule, is presented, confirming the chemical structure of the obtained product.

In the region of the ^1^H NMR spectrum comprised between 9.0 and 6.0 ppm can clearly be seen the three groups of signals belonging to the protons of the benzoxazine, quinoline, and furan moieties. The three signals belonging to the quinoline ring where the nitrogen atom is placed are seen at 8.92, 8.10, and 7.41 ppm, corresponding to H_II_, H_IV_, and H_III_, respectively. All three signals present a *dd* multiplicity, with H_II_ exhibiting both *ortho* and *meta* coupling constants, H_IV_ *meta* and *ortho* coupling constants, and H_III_ *ortho* and *ortho* coupling constants. The four signals assigned to the furfurylamine moiety can be grouped as those on the furan ring, seen at 7.43, 6.34, and 6.27 ppm, corresponding to H_e_, H_d_, and H_c_, whereas that of the methylene group between the nitrogen atom and the furan ring, which is H_a_, is observed at 4.02 ppm. Signals for H_a_, H_c_, H_d_, and H_e_ are present as a singlet, doublet, doublet–doublet, and doublet, respectively. Finally, the signals related to the benzoxazine nucleus are observed at 7.35, 7.14, 5.20, and 4.18 ppm assigned to H_6_, H_5_, H_2_, and H_4_, respectively. While H_6_ and H_5_ are doublets present in the aromatic region, H_2_ and H_4_ are the two typical methylene groups forming the characteristic oxazine ring and present as singlets. The separation between these two signals is 1.02 ppm, which is greater than in the majority of ordinary benzoxazines that exhibit this separation within 0.9–0.6 ppm range [45].

^13^C NMR spectrum complemented this assignment. The most important information concerning the structure elucidation is related to the signals of the typical methylene group forming the oxazine ring and that between the nitrogen atom and the furan ring, C_2_, C_4_, and C_a_, respectively. These carbon signals are observed in Figure 2b at 82.92, 49.65, and 48.66 ppm, respectively.

**Figure 3:**
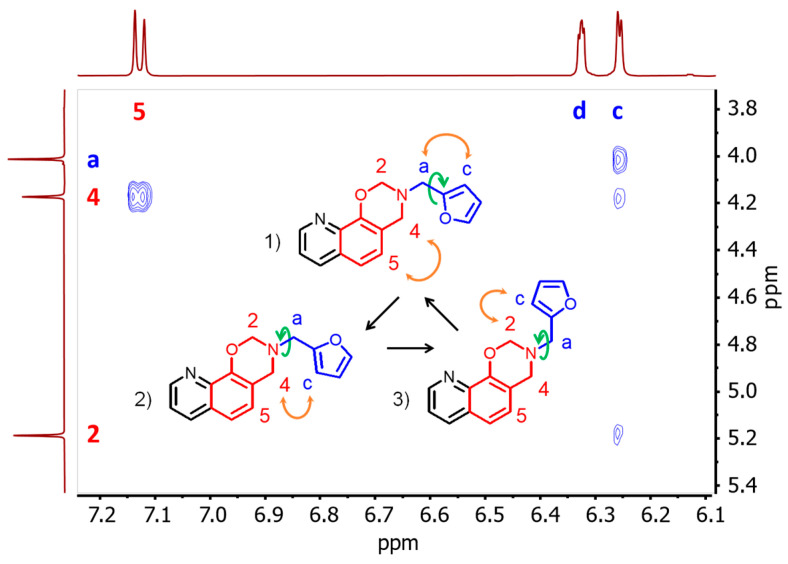
Two-dimensional ^1^H–^1^H NOESY spectrum of **8HQ-fa**. Three possible conformations shown as structures 1, 2, and 3.

The close proximity in the chemical shift in both signal systems, H_4_ and H_a_ on the one hand and C_4_ and C_a_ on the other hand, motivated us to carry out a deeper spectroscopic analysis to unambiguously assign the right signals to the correct chemical structure. Thus, 2D ^1^H–^1^H nuclear Overhauser effect spectroscopy (NOESY) was first used to obtain the correct correlations between the proton signals, detecting through-space interactions between protons in close proximities. The results were afterward utilized to correlate them to their corresponding carbon atoms by means of ^1^H–^13^C heteronuclear multiple quantum correlation (HMQC) NMR spectroscopy. This technique matches directly bonded ^13^C and ^1^H nuclei. Figure 3 shows the 2D ^1^H–^1^H NOESY spectrum of **8HQ-fa**.

As H_6_ and H_5_ are already easily identified by ^1^H NMR (Figure 2b), they are used as starting points to interpret the NOESY NMR spectrum. The first piece of evidence is obtained from the clear through-space interaction between H_5_ and that exhibiting a signal at 4.18 ppm, highlighted in pink. This proton, in turn, presents a through-space interaction with H_c_ (observed at 6.27 ppm), highlighted in orange. These correlations strongly suggest that the signal observed at 4.18 ppm corresponds to H_4_, while that at 4.02 ppm corresponds to H_a_. A complementary piece of evidence is also obtained by understanding that **8HQ-fa** bears two single C-C bonds with free rotation. For instance, in the representation shown in Figure 3 (1), H_4,_ and H_c_ seem to be far from one another. However, after a free rotation over the single C-C bond between C_a_ and C_b_, which is on the furan ring, it is straightforwardly seen that H_4_ and H_c_ are close enough to experience NOE (Figure 3 (2)). Similarly, in the two previous representations, H_c_ and H_2_ seem to be too far away from each other. Once again, after a free rotation now over the single N-C bond between the nitrogen atom and C_a_, it can be seen that H_2_ and H_c_ are also close enough to experience NOE (Figure 3 (3)). This through-space interaction is indeed observed in the NOESY spectrum, as a cross-peak between the mentioned signals. These correlations, in fact, confirm the previous assignments by NOESY NMR spectroscopy stated earlier in this paragraph.

Having unambiguously assigned the proton signals, they are now used as references to interpret the ^1^H–^13^C HMQC NMR spectra (Figure 2d) and correctly assign the carbon signals to the right chemical structure. The correlations between the assigned protons, that is H_2_, H_4_, and H_a_, and the carbon atoms to which they are covalently bonded are shown in Figure 2d. Thus, C_2_, C_4_, and C_a_ are unambiguously assigned to those observed at 82.92, 49.65, and 48.66 ppm, respectively.

### 3.3. Thermal Behavior of Quinolinoxazine Monomer ***8HQ-fa***

**Figure 4:**
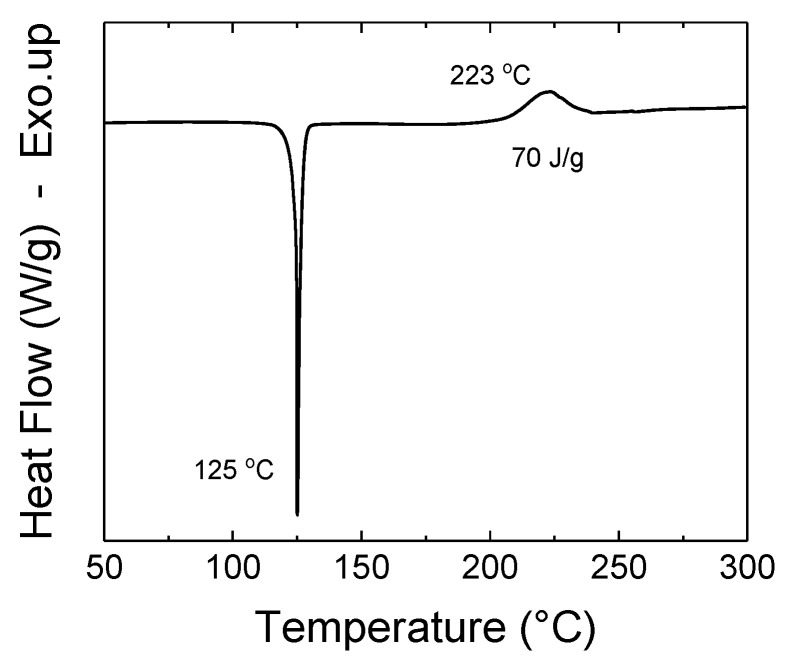
DSC thermogram of the quinolinoxazine monomer, **8HQ-fa**.

Having fully elucidated the chemical structure of the quinolinoxazine monomer synthesized, **8HQ-fa**, the next step was to study its thermal behavior toward polymerization. This was performed by means of DSC (Figure 4).

As can be observed in Figure 4, there are only two noticeable thermal events in the DSC thermogram. The first one corresponds to a truly sharp endothermic event at 125 °C, exhibiting an actual half-width of 1.5 °C for samples of about 2 mg. The second thermal event is present as an exothermic peak with its onset and maximum temperatures at 207 and 223 °C, respectively.

**Figure 5:**
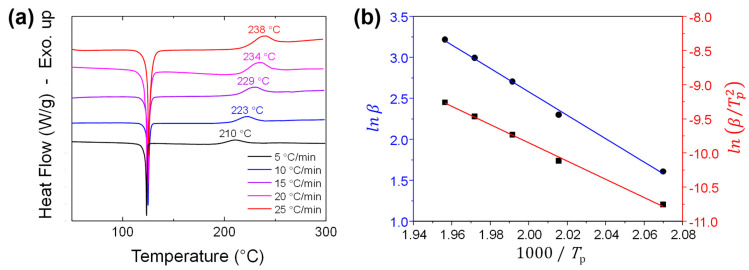
(**a**) DSC thermograms of the quinolinoxazine monomer, **8HQ-fa**, at five different heating rates. (**b**) Plots obtained applying the Kissinger and modified Ozawa methods to graphically calculate the activation energy (*E_a_*) of polymerization of **8HQ-fa**.

The endothermic peak observed at 125 °C in Figure 4 is assigned to the melting of the short needle-like crystals of **8HQ-fa**. The very narrow and reproducible melting signal obtained for this thermal event demonstrates the high purity of the benzoxazine synthesized, which in turn reflects that the fairly low polymerization temperature of **8HQ-fa** is not induced by impurities but rather intrinsic to this monomer. Having anticipated this, the exothermic peak observed at 223 °C in the figure is attributed to the polymerization of **8HQ-fa**. It is also important to notice that between the endothermic melting and exothermic polymerization, there is a broad temperature gap, which could potentially be taken into consideration since it might offer a wide processing window. The uniform shape of the exotherm suggests that the polymerization process might follow a single polymerization mechanism. This motivated us to further study this process by calculating the activation energy (*E_a_*) of the polymerization reaction of **8HQ-fa**. To achieve this goal, the maximum temperature values of each polymerization exotherm measured by DSC as a function of the heating rate were plotted according to the Kissinger [42] and the modified Ozawa [43,44] methods. DSC thermograms at the heating rates of 5, 10, 15, 20, and 25 °C/min and the plots to graphically calculate the *E_a_* of polymerization are shown in Figure 5. Complementary, the Kissinger and modified Ozawa equations are expressed as Equations (1) and (2).

(1) $$ln\left(\frac{\beta }{T_{p}^{2}}\right)=ln\left(\frac{AR}{E_{a}}\right)-\frac{E_{a}}{RT_{p}}\;\;\;\mathrm{Kissinger}\mathrm{equation}$$

(2) $$\ln\beta =-1.052\;\frac{E_{a}}{RT_{p}}+C\;\;\;\mathrm{modified}\mathrm{Ozawa}\mathrm{equation}$$

where *β* is the heating rate, *T_p_* the maximum value of the exothermic polymerization peak, *A* the frequency factor, *R* the gas constant, and finally *E_a_* the activation energy of polymerization.

The results presented in Figure 5b exhibit straight lines in both plots, that is, following the Kissinger and the modified Ozawa method, with slopes of −13.35 and −14.31, respectively. Thus, the *E_a_* for the polymerization reaction of **8HQ-fa** is directly calculated by introducing those slope values into the Kissinger and modified Ozawa equations, resulting in 110.9 and 113.1 kJ/mol, respectively. These values are comparable to other *E_a_* of polymerization of other benzoxazines reported in the literature [36,46,47]. Both plots in Figure 5b, *ln (β/*$\;T_{p}^{2}$*)* and *ln β* as a function of *1/T_p_*, are linear, indicating that **8HQ-fa** might polymerize through a single polymerization mechanism [42,43,44]. This result supports the preliminary observation by DSC for a single exothermic process.

**Figure 6:**
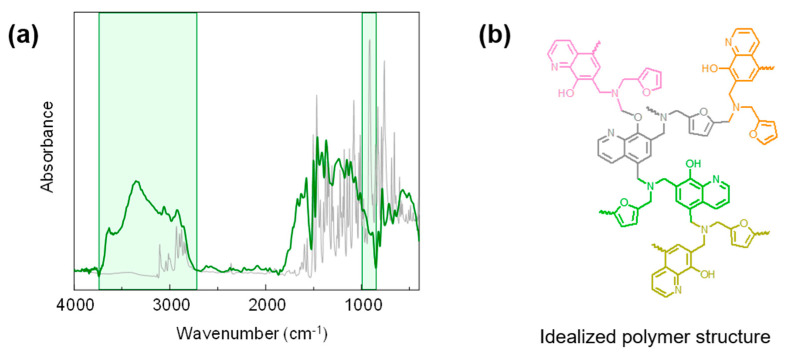
(**a**) FT-IR spectra of **8HQ-fa** (spectrum in gray) and **poly(8HQ-fa)** (spectrum in green). (**b**) Idealized chemical structure of **poly(8HQ-fa)**.

The activation energies for polymerization observed are rather small, indicating the possibility of an intramolecular catalytic polymerization mechanism. Although there appears to be no obvious catalytic group interacting with the oxazine moiety, this might not be the case if one takes into consideration the accepted hypothesis of the oxazine dynamic ring-opening-closing mechanism [1]. If the oxazine ring opens briefly and the oxygen atom takes a hydrogen becoming a phenolic –OH, this hydroxyl group might form an intramolecular 5-membered ring hydrogen bond with the nitrogen atom in the pyridinic ring, thus stabilizing the opened form of the oxazine ring. This, in turn, leaves the reactive iminium anion exposed for longer time, which might increase the reaction rate of polymerization. Although more detailed studies are needed to verify this hypothesis, the combination of the results obtained and discussed hitherto, fairly low polymerization temperature, monomer purity, and polymerization through a single mechanism, is in agreement with an intrinsic and self-initiating polymerization mechanism upon heating [1]. Thus, a schematic idealization of the chemical structure of the resulting crosslinked polymer, **poly(8HQ-fa)**, based on the FT-IR spectroscopy results, is presented in Figure 6. The comparison between the FT-IR spectra of the monomer and the polymer is shown in Figure 6a. It can be seen that the characteristic absorption bands corresponding to the benzoxazine monomer, such as that of the oxazine ring mode observed at 916 cm^−1^, disappeared, while those belonging to the polymer, the phenolic hydroxyl absorption bands between 2900 and 3700 cm^−1^, for instance, appeared. These results indicate a successful polymerization of the monomer and support the idealized chemical structure proposed for the **poly(8HQ-fa)** (Figure 6b).

### 3.4. Thermal Behavior of **Poly(8HQ-fa)**

#### 3.4.1. DSC and TGA Analyses

**Figure 7:**
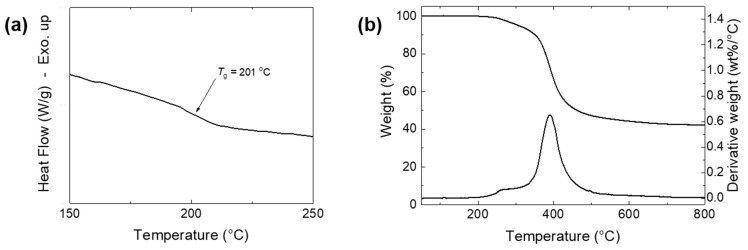
(**a**) DSC and (**b**) TGA thermograms of **poly(8HQ-fa)**.

With the interest in assessing the herein synthesized quinolinoxazine monomer as a sustainable resin to produce a thermally stable thermoset with self-extinguishing properties, **8HQ-fa** was polymerized. As described in the Experimental Section, the polymerization was carried out following a three-step process, thus producing **poly(8HQ-fa)**. The previous DSC results were used to set the polymerization condition, which consisted of three isothermal heating stages: (1) at the exotherm onset (207 °C) for 3 h; (2) at the exotherm maximum (223 °C) for 3 h; (3) at the exotherm endset (239 °C) for 3 h. The thermoset thereof obtained was first characterized by DSC and TGA, and their results are shown in Figure 7.

The DSC thermogram shown in Figure 7a of **poly(8HQ-fa)** exhibits only one thermal event, seen as a *T_g_* at 201 °C. The TGA thermogram shown in Figure 7b presents the weight loss of **poly(8HQ-fa)** as a function of temperature. The DTGA shows that the first degradation step presents a maximum at 272 °C, while the second and most prominent one occurs at 391 °C. The third process is weak and very broad ranging from 504 to 600 °C. Finally, no more significant weight loss seems to occur when exceeding 600 °C. Due to its extreme broadness, the intensity is not obviously observed. This might be due to the broad structural distribution. The thermal event at the lowest temperature is considered to be caused by the loss of the chain ends and/or side groups. Since **poly(8HQ-fa)** is derived from a mono-oxazine functional quinolinoxazine, despite some potential chemical reactivity of the furan ring from the furfurylamine moiety, it might partially contribute to additional crosslinking. Thus, some furan groups might be present as dangling chain ends, which might degrade at this low temperature. The second thermal event is likely due to the cleavage of the main-chain Mannich bridges. The final thermal event is possibly caused by the degradation of the reacted quinoline fused rings that might have a broad structural distribution.

Depending on the targeted application in which the designed thermoset is intended to be used, the thermal stability might be assessed differently. This is one of the reasons why different values are typically reported, such as *T*_d5_, *T*_d10_, and char yield, as shown and defined in Table 3. For instance, a polybenzoxazine based on eugenol and furfurylamine (poly(E-fa)) was reported to exhibit a char yield of 56% [5], suggesting a higher thermal stability than the herein presented **poly(8HQ-fa)**, which exhibited 42%. Nevertheless, this same poly(E-fa) might also be considered as less thermally stable than **poly(8HQ-fa)**, based on its lower *T*_d5_ and *T*_d10_ values of 304 and 336 °C compared to those of the **poly(8HQ-fa)** of 307 and 351 °C, respectively. Needless to say, the exact same situation might be presented the other way around. A higher thermal stability than that of **poly(8HQ-fa)** can be envisaged for a polybenzoxazine based on cardanol and furfurylamine (poly(C-fa)) [4] when comparing their *T*_d5_ and *T*_d10_ values of 374 and 403 °C to those of the **poly(8HQ-fa)** of 307 and 351 °C, respectively. Nevertheless, **poly(8HQ-fa)** can also be viewed as more thermally stable than poly(C-fa) if only their char yields are compared, 42% against 34%, respectively. Overall, the results obtained for the herein presented **poly(8HQ-fa)** are well within and comparable to those of other polybenzoxazine-based thermosets in terms of their thermal properties [6], with *T*_d5_, *T*_d10_, and char yield values ranging from 288 to 420 °C, from 323 to 467 °C, and from 34 to 65%, respectively. To provide context to these values, the properties of the most commonly studied polybenzoxazine, poly(BA-a) [46] are shown in the table for comparison. The favorable properties of this novel polyquinolinoxazine come with the advantages of room-temperature synthesis of the corresponding monomers complemented with a lower polymerization temperature than the majority of benzoxazines.

#### 3.4.2. Flame Retardancy Based on Limiting Oxygen Index

The fairly good thermal stability observed from the TGA results further motivated us to go one step deeper and analyze **poly(8HQ-fa)** in terms of its flame retardancy behavior. In fact, this study might be simply viewed as an extension of TGA since the limiting oxygen index (LOI) can indeed be mathematically calculated for halogen-free polymers according to the van Krevelen equation (Equation (3)) as follows:

LOI = 17.5 + 0.4 × CY   van Krevelen equation(3)

where LOI stands for limiting oxygen index and CY refers to the char yield measured by TGA at 800 °C under nitrogen atmosphere. As shown in Table 3, the LOI value calculated for **poly(8HQ-fa)** is 34.3. It has been demonstrated that in the absence of factual information, flame retardancy can be evaluated based on the calculated LOI value. For the evaluation, three major considerations are taken into consideration. First, atmospheric air is composed, on average, of 21% oxygen. Second, LOI represents the minimum percentage of oxygen required by halogen-free polymers to undergo normal combustion. And third, based on the two previous statements, a scale of LOI values has been established [48,49] stating that polymers with:

- LOI values < 21 are flammable since they easily burn in air;
- LOI values between 21 and 28 slowly burn;
- LOI values > 28 are self-extinguishing since they cease burning once removed from the ignition source.

Based on the obtained result, a LOI value of 34.3 for **poly(8HQ-fa)**, this polybenzoxazine is classified as an effective self-extinguishing thermoset.

#### 3.4.3. Microscale Combustion Calorimetry

**Figure 8:**
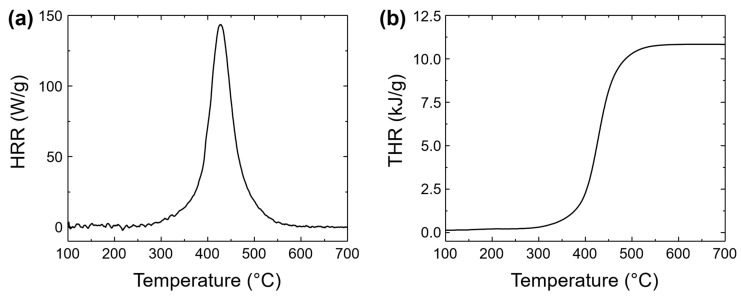
Plots of heat release rate (**a**) and total heat release (**b**) as a function of temperature for **poly(8HQ-fa)**.

The combination of the previous results awoke us a natural interest in assessing the flame-retardant properties of **poly(8HQ-fa)**. Nevertheless, this time, the assessment is based on factual information, generated by means of microscale combustion calorimetry. Figure 8 shows the thermograms obtained as plots of heat release rate (Figure 8a) and total heat release (Figure 8b) as a function of temperature for **poly(8HQ-fa)**.

As can be seen in Figure 8a, **poly(8HQ-fa)** burns in the temperature region between about 300 and 550 °C, exhibiting a heat release capacity (HRC) of 143 J/gK. While the complementary result, total heat release (THR), is shown in Figure 8b, exhibiting a value of 10.8 kJ/g, the fire growth capacity (FGC), which is calculated upon the measured THR, is 116 J/g-K. The relatively narrow thermogram shown in Figure 8a is due to the polymer being derived from a mono-oxazine precursor, producing a thermoset with a moderate crosslinking density.

In order to set these values into context, as in the case of LOI, there is a simple manner of classification for polymers with the following traits:

- HRC values > 300 J/gK are flammable;
- HRC values < 300 J/gK are considered self-extinguishing;
- HRC values < 100 J/gK are considered nonignitable.

Based on the obtained result, a HRC value of 143 J/gK for **poly(8HQ-fa)**, this polybenzoxazine is classified as an effective self-extinguishing thermoset.

## 4. Conclusions

A novel quinoline-containing benzoxazine resin, **8HQ-fa**, was designed and efficiently synthesized using 8-hydroxyquinoline and furfurylamine as the phenol and amine source, respectively. Since the phenolic compound can be obtained from waste, the amine indirectly produced from natural renewable sources, and the synthesis carried out at room temperature, the synthesis can be considered as sustainable. **8HQ-fa** exhibited a melting point of 125 °C and a fairly low polymerization temperature of 223 °C, presenting a broad temperature gap, thus offering a wide processing window. The resulting thermoset, **poly(8HQ-fa)**, was shown to bear good thermal properties as evidenced by its *T_g_* (201 °C), *T*_d5_, and *T*_d10_ (307 and 351 °C, respectively) and char yield (42%). Interestingly, **poly(8HQ-fa)** also exhibited good anti-flammable properties, such as a LOI value (34.3) significantly greater than the established value for polymers to be considered as self-extinguishing (28); a heat release capacity value (143 J/gK) well within the range set for polymers is considered as self-extinguishing (100–300 J/gK). Last but not least, as in the case of the recognized naphthozaxines, we propose the use of quinolinoxazine for quinoline-based or quinoline-containing benzoxazines.

## Figures and Tables

**Table 1:** Reaction conditions for the synthesis of **8HQ-fa**.

Reaction	Quinoline	Amine	Formaldehyde Source	Acid	Reaction Yield (%)
1	8HQ	fa	paraformaldehyde	-	14
2	8HQ	fa	paraformaldehyde	HCOOH	6
3	8HQ	fa	formalin	-	80
4	8HQ	fa	formalin	HCOOH	87

**Table 2:** List of assigned bands indicating the successful formation of **8HQ-fa**.

Assigned FT-IR Signal Observed in Spectrum a (cm^−1^)	Assignment
1246	antisymmetric stretching of =C-O-C-
1180	antisymmetric stretching of C-N-C
1126	benzene ring mode, Wilson number 15
1080	antisymmetric stretching of C-O-C and C-N-C
1028	symmetric stretching of =C-O-C-
916	oxazine ring mode
853	symmetric stretching of C-O-C

**Table 3:** Thermal Properties of thermoset, **poly(8HQ-fa)**.

Thermoset	*T*_g_ (°C) ^a^	*T*_d5_ (°C) ^b^	*T*_d10_ (°C) ^c^	Char Yield (%) ^d^	LOI ^e^	HRC (J g^−1^ K^−1^) ^f^	THR (kJ g^−1^) ^g^
**Poly(8HQ-fa)**	201	307	351	42	34.3	143	10.8
**Poly(BA-a)**	162	302	324	32	30.3	328	35.3

^a^ Glass transition temperature. ^b^ Temperature at which the weight loss corresponds to 5%. ^c^ Temperature at which the weight loss corresponds to 10%. ^d^ Char yield, defined as the remaining weight% at 800 °C, measured under N_2_. ^e^ LOI is limiting oxygen index. ^f^ HRC is heat release capacity. ^g^ THR is total heat released.

## Data Availability

The original contributions presented in this study are included in the article.
